# Mitochondrial protection by the mixed muscarinic/σ_1_ ligand ANAVEX2-73, a tetrahydrofuran derivative, in Aβ_25–35_ peptide-injected mice, a nontransgenic Alzheimer’s disease model

**DOI:** 10.3389/fncel.2014.00463

**Published:** 2015-01-20

**Authors:** Valentine Lahmy, Romain Long, Didier Morin, Vanessa Villard, Tangui Maurice

**Affiliations:** ^1^Inserm U 710, University of Montpellier 2Montpellier, France; ^2^Amylgen, Montferrier-sur-LezFrance; ^3^Inserm U 955, Team 03, CréteilFrance; ^4^Faculty of Medicine, Université Paris-Est, Unité Mixte de Recherche S955, Université Paris-Est Créteil Val-de-MarneCréteil, France

**Keywords:** Alzheimer’s disease, mitochondrial damages, ANAVEX2-73, sigma-1 receptor, cytoprotection

## Abstract

Alzheimer’s disease (AD), the most prevalent dementia in the elderly, is characterized by progressive synaptic and neuronal loss. Mitochondrial dysfunctions have been consistently reported as an early event in AD and appear before Aβ deposition and memory decline. In order to define a new neuroprotectant strategy in AD targeting mitochondrial alterations, we develop tetrahydro-N,N-dimethyl-2,2-diphenyl-3-furanmethanamine (ANAVEX2-73, AE37), a mixed muscarinic receptor ligand and a sigma-1 receptor (σ_1_R) agonist. We previously reported that ANAVEX2-73 shows anti-amnesic and neuroprotective activities in mice injected intracerebroventricular (ICV) with oligomeric amyloid-β_25–35_ peptide (Aβ_25–35_). The σ1R is present at mitochondria-associated endoplasmic reticulum (ER) membranes, where it acts as a sensor/modulator of ER stress responses and local Ca^2+^ exchanges with the mitochondria. We therefore evaluated the effect of ANAVEX2-73 and PRE-084, a reference σ_1_R agonist, on preservation of mitochondrial integrity in Aβ_25–35_-injected mice. In isolated mitochondria from hippocampus preparations of Aβ_25–35_ injected animals, we measured respiration rates, complex activities, lipid peroxidation, Bax/Bcl-2 ratios and cytochrome *c* release into the cytosol. Five days after Aβ_25–35_ injection, mitochondrial respiration in mouse hippocampus was altered. ANAVEX2-73 (0.01–1 mg/kg IP) restored normal respiration and PRE-084 (0.5–1 mg/kg IP) increased respiration rates. Both compounds prevented Aβ_25–35_-induced increases in lipid peroxidation levels, Bax/Bcl-2 ratio and cytochrome *c* release into the cytosol, all indicators of increased toxicity. ANAVEX2-73 and PRE-084 efficiently prevented the mitochondrial respiratory dysfunction and resulting oxidative stress and apoptosis. The σ_1_R, targeted selectively or non-selectively, therefore appears as a valuable target for protection against mitochondrial damages in AD.

## Introduction

Alzheimer’s disease (AD), the most common form of dementia in the elderly is defined histologically by the presence of the two hallmarks: extracellular senile plaques constituted with amyloid-β (Aβ) proteins and intracellular inclusions of hyperphosphorylated microtubule-associated Tau protein, refered as neurofibrillary tangles (Selkoe, 2004). Clinically, the disease is characterized by progressive cognitive decline associated with synaptic and neuronal loss in regions critical for memory process, *e.g*., hippocampus, entorhinal and frontal cortex (Crews and Masliah, 2010). Mitochondria, which are the main energy provider in the cell, are particularly enriched in synapses where energy is critical for synaptic transmission (Verstreken et al., 2005). Mitochondrial dysfunctions have been consistently reported as an early event in AD physiopathology and appear before Aβ deposition and memory deficits in AD patients and transgenic mice (Maurer et al., 2000; Caspersen et al., 2005; Mosconi et al., 2008). Indeed, during AD pathogenesis, Aβ oligomers accumulate in mitochondria, resulting in disrupted energy metabolism, increased oxidative stress and apoptosis (Lustbader et al., 2004; Caspersen et al., 2005; Manczak et al., 2006). Studies in AD patients and transgenic mice have reported consistent decreases in tricarboxylic acid cycle enzymes and cytochrome *c* oxidase activities (Yates et al., 1990; Maurer et al., 2000; Caspersen et al., 2005; Leuner et al., 2007). Moreover, studies in isolated mitochondria from transgenic mice brains or in isolated mitochondria exposed to Aβ oligomers showed decreased respiration rates and ATP production and increased oxidative stress, suggesting Aβ-related defects in mitochondrial respiratory chain (Casley et al., 2002a; Aleardi et al., 2005; Caspersen et al., 2005; Clementi et al., 2005; Leuner et al., 2007). Disruption in mitochondrial respiratory chain results in increased production of oxidative stress by mitochondria, a key element in AD physiopathology (Smith et al., 1996; Leuner et al., 2012). One other aspect of mitochondrial failure is the opening of mitochondrial permeability transition pore (mPTP) which can be triggered by different effectors, *e.g*., mitochondrial Ca^2+^ overload and oxidative stress (Kroemer and Reed, 2000). mPTP opening could also be triggered by the translocation to the mitochondrial outer membrane of the cytosolic proapototic members of the Bcl-2 family proteins. Among them is the proapototic protein Bax which, in proapoptotic conditions, translocates to mitochondria, dimerizes and constitutes a pore in the mitochondrial membrane and/or contributes to the formation of mPTP (Jürgensmeier et al., 1998; Marzo et al., 1998; Narita et al., 1998). Bax activity could be antagonized by heterodimerization with antiapoptotic Bcl-2 family proteins, including Bcl-2 itself (Hanada et al., 1995; Yin et al., 1995; Yang et al., 1997). mPTP opening allows liberation of mitochondrial protein, such as cytochrome *c*, which promotes apoptosis, binding to the apoptosis protease activation factor (APAf-1) and forming a complex indicated as “apoptosome” (Liu et al., 1996; Yang et al., 1997).

In the present study, we used the intracerebroventricular (ICV) injection of Aβ_25–35_ fragment in oligomeric form in mice as a rapid, standardized pharmacological model of AD toxicity, suitable to investigate the impact of mitochondrial alteration. Indeed, ICV Aβ_25–35_ injection results in neuroinflammation and reactive gliosis, pro-apoptotic caspase activity, oxidative stress, reduction in the number of neurons measured in hippocampal pyramidal cell layers, loss of cholinergic neurons, and memory deficits (Maurice et al., 1996; Delobette et al., 1997; Meunier et al., 2006; Villard et al., 2009, 2011; Zussy et al., 2011). *In vitro* evidences have been accumulated suggesting that the peptide also altered mitochondrial physiology, after direct application on isolated mitochondria or in mitochondria isolated from cultured neurons exposed to Aβ_25–35_ (Canevari et al., 1999; Casley et al., 2002a,b; Aleardi et al., 2005; Clementi et al., 2005; Dong et al., 2010; Ren et al., 2011). Isolated mitochondria from rat brain subjected to subchronic ICV injection of Aβ_25–35_ or Aβ_1–40_ produced significantly more H_2_O_2_ compared to control mice after three days of treatment, suggesting mitochondrial dysfunction in this model (Kaminsky and Kosenko, 2008). However, to our knowledge, the functionality of isolated mitochondria has never been explored *ex vivo* after ICV administration of Aβ_25–35_ in rodents.

Tetrahydro-N,N-dimethyl-2,2-diphenyl-3-furanmethanamine hydrochloride (ANAVEX2-73, AE37) is a novel ligand, acting at muscarinic acetylcholine (mAChR) and σ_1_ receptors (σ_1_R) with affinities in the low micromolar range (Espallergues et al., 2007). We previously reported that this compound had anti-amnesic and neuroproective potential in the AD model in mice induced by the ICV injection of Aβ_25–35_ peptide. In particular, ANAVEX2-73 attenuated cellular loss, Aβ_1–42_ seeding, Tau hyperphosphorylation and learning and memory deficits observed several days after Aβ_25–35_ injection (Villard et al., 2009, 2011; Lahmy et al., 2013). The drug acts synergistically on its two main targets, as an antagonist of M2 mAChR and as an agonist of σ_1_R. The σ_1_R is an endoplasmic reticulum (ER)-resident molecular chaperone (Hayashi and Su, 2007), also expressed at the mitochondrial membrane (Klouz et al., 2002), particularly at mitochondria-associated ER membranes (MAM). Its activity regulates Ca^2+^ exchange from ER to mitochondria (Hayashi and Su, 2007), known to be critical for mitochondrial bioenergetics (Cárdenas et al., 2010). Moreover, the σ_1_R is involved in regulation of the mitochondrial membrane potential, reactive oxygen species (ROS) production and apoptosis (Tsai et al., 2009; Meunier and Hayashi, 2010) and σ_1_R agonists have been shown to protect mitochondrial function *in vivo* against ischemic damage (Klouz et al., 2008; Tagashira et al., 2013).

In this study, we analyzed whether the neuroprotective activity of ANAVEX2-73 involves mitochondrial protection. We analyzed the respiratory activity in mitochondria isolated from the mouse hippocampus, five days after ICV injection of oligomerized Aβ_25–35_. We measured respiration rates of freshly extracted mitochondria and the activity of the respiratory chain complexes I to IV. We then analyzed biochemical markers of mitochondrial damage, including Bax, Bcl-2 protein expression and cytochrome *c* release from the mitochondria into the cytosol. PRE-084 was used as a reference σ_1_R agonist.

## Material and methods

### Animals

Male Swiss OF-1 mice (Depré, St Doulchard, France), aged 7–9 weeks and weighing 32 ± 2 g were used. They were housed in plastic cages in groups with free access to food and water, except during behavioral experiments. They were kept in a regulated environment (23 ± 1°C, 50–60% humidity) under a 12 h light/dark cycle (light on at 8:00 am). Behavioral experiments were carried out between 10:00 am and 4:00 pm, in an experimental room within the animal facility. All animal procedures were conducted in adherence with the 2010/63 EU Directive.

### Drugs and administration procedures

The amyloid-β[25–35] (Aβ_25–35_) and scrambled Aβ_25–35_ (Sc. Aβ) peptides were purchased from Genepep (Saint Jean-de-Védas, France). They were solubilized in sterile distilled water at a concentration of 3 mg/ml and stored at −20°C until use. Before injection, peptides were aggregated by incubation at 37°C for 4 days (Maurice et al., 1996). This procedure is currently standardized in the laboratory and previous analyses using electronic microscopy, IR spectroscopy, and photon correlation spectroscopy techniques showed that the aggregated Aβ_25–35_ solution is mainly composed of a mixture of soluble oligomeric amyloid species, which sizes extended from 52.8 to 295.3 nm (98%) (Zussy et al., 2011, 2013). They were administered intracerebroventricularly (ICV) in a final volume of 3 µl per mouse, as previously described (Maurice et al., 1996; Meunier 2006). Tetrahydro-N,N-dimethyl-2,2-diphenyl-3-furanmethanamine hydrochloride (ANAVEX2-73, AE37) was provided by Dr Alexandre Vamvakides (Anavex Life Science, Pallini, Greece). PRE-084 was a gift from Dr Tsung-Ping Su (NIDA/NIH, Baltimore, MD, USA). 2-(3,4-Dichlorophenyl)-N-(2-dimethylaminoethyl)-N-methylethanamine dihydrobromide (BD1047) was from Sigma-Aldrich (St Quentin-Fallavier, France). Drugs were solubilized in physiological saline at the concentration of 5 mg/ml and then diluted to the appropriate concentrations. They were injected intraperitoneally (IP) in a volume of 100 µl/20 g body weight. Drugs were injected once, 20 min before the Aβ_25–35_ peptide, also injected once. Animals were used 1, 3, 5, or 7 days after injections, as indicated in the figure legends.

### Lipid peroxidation

Mice were killed by decapitation and brains were rapidly removed, the hippocampus dissected out, weighed, and kept in liquid nitrogen until assayed. After thawing, the hippocampus was homogenized in cold methanol (1/10 w/v), centrifuged at 1,000 g for 5 min and the supernatant collected. Homogenate was added to a solution containing 1 mM FeSO_4_, 0.25 M H_2_SO_4_, 1 mM xylenol orange, and incubated for 30 min at room temperature. Absorbance was measured at 580 nm (A_580_1), and 10 ml of 1 mM cumene hydroperoxide (CHP; Sigma-Aldrich) was added to the sample and incubated for 30 min at room temperature, to determine the maximal oxidation level. Absorbance was measured at 580 nm (A_580_2). The level of lipid peroxidation was determined as CHP equivalents according to: CHP eq. 1/4 A_580_1/A_580_2 [CHP (nmol)] dilution, and expressed as CHP eq. *per* wet tissue weight.

### Respiration rates

Five days after peptide injection, mice were sacrificed, and their hippocampus rapidly removed on ice and homogenized in 10 ml of ice-cold homogenization buffer (220 mM mannitol, 70 mM sucrose, 10 mM 2-[4-(2-hydroxyethyl)piperazin-1-yl]ethanesulfonic acid (HEPES; Sigma-Aldrich), 2 mM ethylene glycol tetraacetic acid (EGTA; Sigma-Aldrich), pH 7.4) using a Teflon potter homogenizer. Homogenate was centrifuged at 1,000 g for 5 min. The supernatant was then centrifuged at 10,000 g for 10 min. Pellet was collected and resuspended in 50 µl of ice-cold isolation buffer (220 mM mannitol, 70 mM sucrose, 10 mM HEPES, 0.01 mM EGTA, pH 7.4). Mitochondrial protein content was determined by spectrophotometry. O_2_ consumption was measured with a Clarke type oxygen electrode (Hansatech, Cergy, France). Mitochondria (0.8 mg/ml) were loaded in the chamber filled up with respiration buffer maintained at 30°C (50 mM sucrose, 100 mM KCl, 10 mM HEPES, 5 mM KH_2_PO_4_, 0.5 mM MgCl_2_, pH 7.4). State 2 respiration was measured after addition of 5 mM pyruvate-malate and state 3 respiration was triggered by addition of 0.5 mM adenosine diphosphate (ADP; Sigma-Aldrich). State 4 respiration was then measured after addition of 4 µM carboxyatractyloside (CAT; Sigma-Aldrich), a blocker of the adenosine trisphosphate (ATP)-ADP carrier, and uncoupled state with 1 µM tyrphostine (uncoupling agent). The mean of two measures of respiration rates for the four states were calculated for each sample. The control ratio was calculated as the ratio between state 3 and state 4 respiratory rates.

In a series of experiment, the effects of direct application of the drugs (ANAVEX2-73 and PRE-084, in the 10^−8^ to 10^−4^ M concentration range) were examined. Isolated mitochondria (0.8 mg/ml) were loaded in the chamber. Drugs were applied and after 5 min, the respiration was examined.

### Complex activities

Five days after peptide injection, mice were sacrificed, and their hippocampus rapidly removed on ice. Mitochondria were isolated in the same way as for respiration rates. Mitochondrial protein content was determined using a BCA assay (Pierce Biotechnology, Rockford, IL, USA). Mitochondria were frozen/thawed four times to break mitochondrial membranes. Complex activities were measured at 30°C, using a Jasco V-530 spectrophotometer (Jasco, Nantes, France). Complex activities are calculated as nmol/min/mg of proteins and expressed as percentage of Sc. Aβ-treated group data.

#### Complex I (NADH ubiquinone reductase)

The oxidation of nicotinamide adenine dinucleotide (NADH; Sigma-Aldrich) by complex I was measured using decylubiquinone (DUQ; Sigma-Aldrich), an ubiquinone analog, as electron acceptor. 200 µM NADH and 100 µM DUQ were added to the assay medium (25 mM KH_2_PO_4_, 5 mM MgCl_2_, 250 µM KCN, 1 mg/ml bovine serum albumin (BSA; Sigma-Aldrich), pH 7.4) in a final volume of 1.5 ml. Enzyme activity was measured by initiating the reaction with 0.15 mg of mitochondrial protein. The decrease in absorbance due to NADH oxidation was measured at 340 nm.

#### Complex II (succinate dehydrogenase)

The activity of complex II was measured indirectly by record of the reduction of 2,6-dichlorophenolindo-phenol (DCIP; Sigma-Aldrich) following the oxidation of succinate. 6 mM succinate was incubated with 0.05 mg of mitochondrial proteins for 5 min at 30°C in the assay medium (10 mM KH_2_PO_4_, 2 mM ethylenediaminetetraacetic acid (EDTA; Sigma-Aldrich), 2 µM rotenone, 1 mg/ml BSA) in a final volume of 1.5 ml. 100 µM DUQ and 80 µM DCIP were then added and the decrease in absorbance at 600 nm, corresponding to the reduction of DCIP, was measured.

#### Complex III (ubiquinol cytochrome *c* reductase)

The oxidation of DUQH_2_ by complex III was determined using cytochrome *c*(III) as an electron acceptor. 0.25 mg of mitochondrial protein was added in the assay medium (10 mM KH_2_PO_4_, 2 mM EDTA, 1 mg/ml BSA, 2 µM rotenone, 250 µM KCN) in a final volume of 1.5 ml. The reaction was started with the addition of 100 µM DUQH_2_ (Sigma-Aldrich) and the increase in absorbance at 550 nm, corresponding to reduction of cytochrome *c* was measured. Because cytochrome *c*(III) could also be reduced by DUQH_2_ independently of complex III, the reduction of cytochrome *c*(III) was measured in the presence of 1 µM antimycin (an inhibitor of complex III activity) and deducted from the total reduction measured before.

#### Complex IV (cytochrome c oxidase)

0.025 mg of mitochondrial protein were incubated for 1 min at 30°C in the assay medium (10 mM KH_2_PO_4_, 10 mM EDTA, 2 mM MgCl_2_, 1 mg/ml BSA) in a final volume of 1.5 ml. The reaction was started when 33 µM of cytochrome *c*(II) was added to the assay medium and the decreased in absorbance at 550 nm, corresponding to oxidation of cytochrome *c*(II), was measured.

### Western blotting

Mice were sacrificed at indicated days after injections and the hippocampus rapidly dissected on ice and kept at −80°C until used. For cytochrome *c* release experiments, the hippocampus were homogenized with a glass Dounce homogenizer in ice-cold homogenization buffer (250 µM sucrose, 10 mM HEPES, pH 7.4), including a protease and phosphatase inhibitor cocktail (Roche Diagnostics, Meylan, France) in a final volume of 250 µl. Homogenates were centrifuged at 600 g for 5 min and the supernatant collected and centrifuged again at 10,300 g for 20 min. The supernatant, corresponding to the cytosolic fraction (C), and the pellet, corresponding to the crude mitochondrial fraction (M), were separated. The mitochondrial fraction was resuspended in 50 µl of ice-cold isolation buffer (250 mM mannitol, 5 mM HEPES, 0.5 mM EGTA, pH 7.4).

For Bax and Bcl-2 experiments, the hippocampus were homogenized by sonication in an ice-cold lysis buffer (125 mM Tris HCl pH 6.8, 4% sodium dodecyl sulfate (SDS; Sigma-Aldrich), 20% glycerol) including a protease and phosphatase inhibitor cocktail (Roche Diagnostics, Meylan, France). Homogenates were heated at 70°C for 10 min and centrifuged at 16,000 g for 30 min. The supernatant was collected.

For all samples, protein concentration was determined using a BCA assay (Pierce Biotechnology, Rockford, IL, USA) according to the manufacturer’s instructions.

Proteins, 20 µg per lane, were resolved on a 12% SDS-polyacrylamid gel and transfered to a polyvinylidene fluoride (PVDF) membrane (GE Healthcare, Orsay, France). After 1 h blocking in 5% non-fat dry milk in a 20 mM Tris-buffered saline pH 7.5 buffer containing 0.1% Tween-20 (TBS-T), membranes were incubated overnight at 4°C with the following primary antibodies: mouse anti-cytochrome c (CytC, dilution 1/1000; BioLegend, San Diego, CA, USA), mouse anti-oxphos-complex IV subunit I (Oxphos, 1/1000; Invitrogen Life Technologies, St Aubin, France), rabbit anti-Bax (1/2000; Cell Signaling Technology, Ozyme, St Quentin-en-Yvelines, France), mouse anti-Bcl-2 antibody (1/1000; Santa Cruz Biotechnology; Heidelberg, Germany). After brief washes, membranes were incubated for 1 h at room temperature with corresponding secondary antibody: goat anti-mouse IgG peroxidase conjugate (1/2000; Sigma-Aldrich) or goat anti-rabbit IgG peroxidase conjugate (1/2000; Sigma-Aldrich). The immunoreactive bands were visualized with the enhanced chemiluminescence reagent (ECL, Millipore, Molsheim, France) using an Odyssey® Fc fluorescent imaging system (Li-Cor, Eurobio, Courtaboeuf, France). Then, membranes were stripped using the Restore Western Blot Stripping Buffer (Pierce Biotechnology) and reprobed with anti-β-tubulin antibody (1/2000) (Sigma-Aldrich). The intensity of peroxidase activity was quantified using the Odyssey® Fc software (Li-Cor).

### Statistical analyses

Data were expressed as mean ± S.E.M. The number of animals is indicated in the figure legends. Data were analyzed using one-way ANOVA (*F* values), followed by the Dunnett’s *post-hoc* multiple comparison test. For reading clarity, ANOVA values were all reported in the figure legends. The level of statistical significance was *p* < 0.05.

## Results

**Figure 1:**
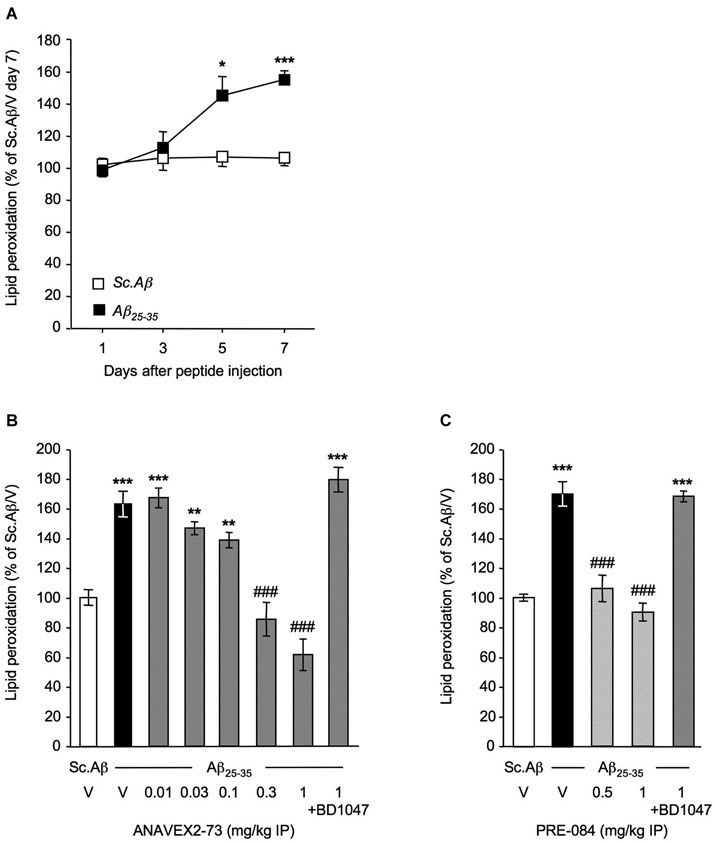
**ANAVEX2-73 and PRE-084 prevented oxidative stress in Aβ_25–35_-treated mice**. Mice were administered ICV with Sc. Aβ or Aβ_25–35_ peptide (9 nmol) and sacrificed after 1, 3, 5 or 7 days for lipid peroxidation measures **(A)**. *n* = 6 per group, ^*^p < 0.05, ^***^p < 0.001 vs. the (Sc. Aβ + V)-treated group at the same timepoint; *t*-test. Mice were then administered IP with **(B)** ANAVEX2-73 (0.01–1 mg/kg) or **(C)** PRE-084 (0.5–1 mg/kg) 20 min before Aβ_25–35_ (9 nmol). In two groups, BD1047 (10 mg/kg) was administered simultaneously with the highest dose of each agonist. Mice were sacrificed after 7 days for lipid peroxidation measures. One-way ANOVA: *F*_(7,70)_ = 22.5, *p* < 0.0001, *n* = 6–14 per group in **(B)**; *F*_(4,69)_ = 31.9, *p* < 0.0001, *n* = 6–22 in **(C)**. ^**^p < 0.01, ^***^p < 0.001 vs. the (Sc. Aβ+V)-treated group; ^###^*p* < 0.001 vs. the (Aβ_25–35_+V)-treated group; Dunnett’s test.

ICV administration of Aβ_25–35_ in mice induced a robust (+50–70%) increase in lipid peroxidation level in the hippocampus of the animals, starting at 5–7 days after injection (Figure 1A). The IP pretreatment with ANAVEX2-73, in the 0.01–1 mg/kg dose-range dose-dependently prevented the Aβ_25–35_-induced increase in lipid peroxidation with highly significant effect measured at 0.3 and 1 mg/kg (Figure 1B). The reference σ_1_R agonist PRE-084 similarly blocked Aβ_25–35_-induced increase in lipid peroxidation at 0.5 and 1 mg/kg (Figure 1C). Note that both drug effects at the highest doses were completely blocked by the reference σ_1_R antagonist BD1047, confirming the σ_1_R selectivity (Figures 1B,C). These initial observations confirmed that ICV injection of Aβ_25–35_ induces a marked oxidative stress in mice and that the two compounds act as anti-oxidant drugs. The prominent role of mitochondrial dysfunction in ROS generation and oxidative stress led us to analyze the impact of the drug treatments on mitochondrial respiration.

**Figure 2:**
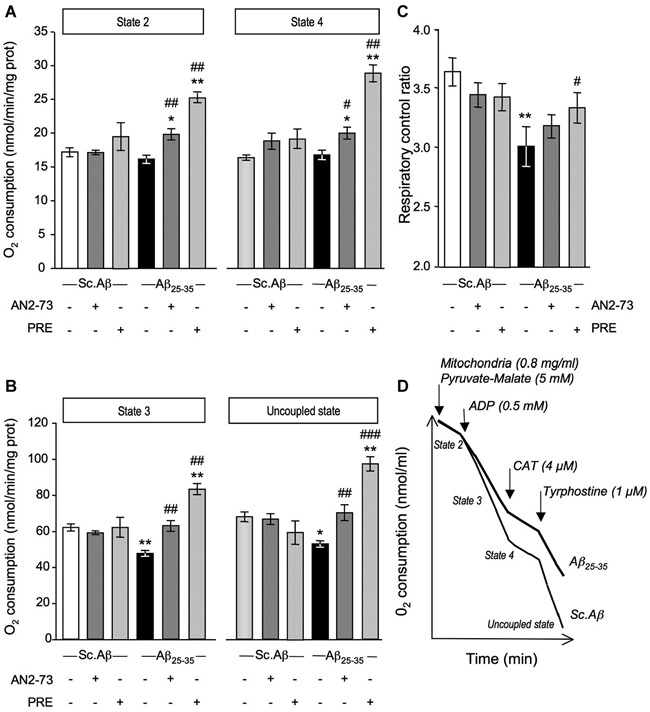
**ANAVEX2-73 (AN2-73) and PRE-084 (PRE) protected against Aβ_25–35_-induced alteration of mitochondrial respiration in mice**. Mice were treated with ANAVEX2-73 (0.3 mg/kg IP), PRE-084 (0.5 mg/kg IP) or vehicle, before injection of Sc. Aβ or Aβ_25–35_ (9 nmol ICV). **(A–C)** Mitochondria (0.8 mg/ml) were loaded in the chamber with appropriate buffer at 30°C. State 2 respiration was activated by addition of pyruvate-malate (5 mM). State 3 respiration was induced with ADP. State 4 respiration was provoked by the addition of carboxyatractyloside (CAT), a blocker of ATP-ADP carrier. Finally, addition of tyrphostine, an uncoupling agent, activated the uncoupled respiration. The respiratory control ratio is the state 3/state 4 ratio. *n* = 4–8 per group, *F*_(5,38)_ = 7.11, *p* < 0.0001 for state 2 and *F*_(5,38)_ = 8.43, *p* < 0.0001 for state 4 in **(A)**;* F*_(5,38)_ = 13.1, *p* < 0.0001 for state 3 and *F*_(5,38)_= 8.43, *p* < 0.0001 for state 3 in **(B)**; *F*_(5,38)_ = 2.58, *p* < 0.05 in **(C)**. ^*^p < 0.05, ^**^p < 0.01 vs. Sc. Aβ/Veh; ^#^*p* < 0.05, ^##^*p* < 0.01, ^###^*p* < 0.001 vs. Aβ_25–35_/Veh. Dunnett’s test. **(D)** Typical trace of O_2_ consumption for Sc. Aβ and Aβ_25–35_-injected mice.

**Figure 3:**
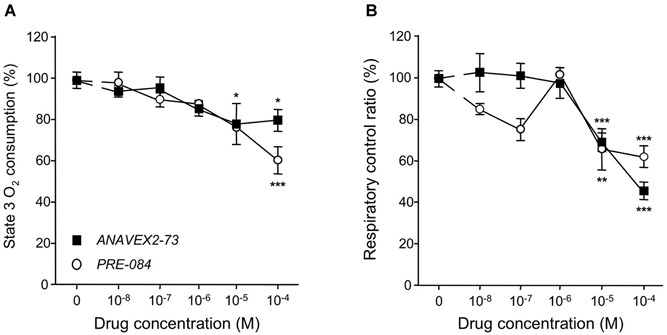
**Direct application of ANAVEX2-73 or PRE-084 affected mitochondrial respiration only at high concentrations**. Mitochondria (0.8 mg/ml) were loaded in the chamber with appropriate buffer at 30°C. The drugs (10^−8^ to 10^−4^ M) were bath applied 5 min before the repspiration measures. State 2 respiration was activated by addition of pyruvate-malate (5 mM). State 3 respiration was induced with ADP. State 4 respiration was provoked by the addition of carboxyatractyloside (CAT), a blocker of ATP-ADP carrier. The respiratory control ratio is the state 3/state 4 ratio. *n* = 4–8 per group (*n* = 14 for the no-drug group), *F*_(5,38)_ = 3.08, *p* < 0.05 for ANAVEX2-73 and *F*_(5,31)_ = 5.19, *p* < 0.01 for PRE-084 in **(A)**;* F*_(5,38)_ = 22.1, *p* < 0.0001 for ANAVEX2-73 and *F*_(5,31)_= 7.53, *p* < 0.001 for PRE-084 in **(B)**. ^*^p < 0.05, ^**^p < 0.01, ^***^p < 0.001 vs. no-drug data; Dunnett’s test.

O_2_ consumption was analyzed in mice, five days after Sc. Aβ and Aβ_25–35_ injection, and following ANAVEX2-73 or PRE-084 treatment (Figure 2). The Aβ_25–35_ ICV treatment failed to affect state 2 or state 4 O_2_ consumption (Figure 2A), but significantly altered by −23% and −22% the state 3 or uncoupled state O_2_ consumption, respectively (Figure 2B). As a consequence the peptide injection significantly altered the respiratory control ratio by −17% (Figure 2C). The ANAVEX2-73 treatment (0.3 mg/kg IP) significantly prevented the Aβ_25–35_-induced decreases in O_2_ consumption (Figure 2B). However, since the drug increased in fact significantly O_2_ consumption at all states (Figure 2A), there was no significant impact on the respiratory control ratio (Figure 2C). A similar profile was observed with PRE-084 (0.5 mg/kg IP) in Aβ_25–35_-treated mice (Figures 2A,B) but the increase in state 3 O_2_ consumption was more marked resulting in a restoration of the respiratory control ratio value,* i.e*., a significant prevention of the Aβ_25–35_-induced alteration (Figure 2C). Note that neither ANAVEX2-73 nor PRE-084 affected O_2_ consumption in Sc. Aβ-treated animals. In addition, we analyzed whether the drugs have a direct effect on mitochondrial respiration. The drugs were both applied at increasing concentrations, from 10^−8^ M to 10^−4^ M, on isolated mitochondria and respiration analyzed. State 3 O_2_ consumption and respiratory control ratio are presented (Figures 3A,B). The drugs affected both parameters only at the highest concentrations tested.

**Figure 4:**
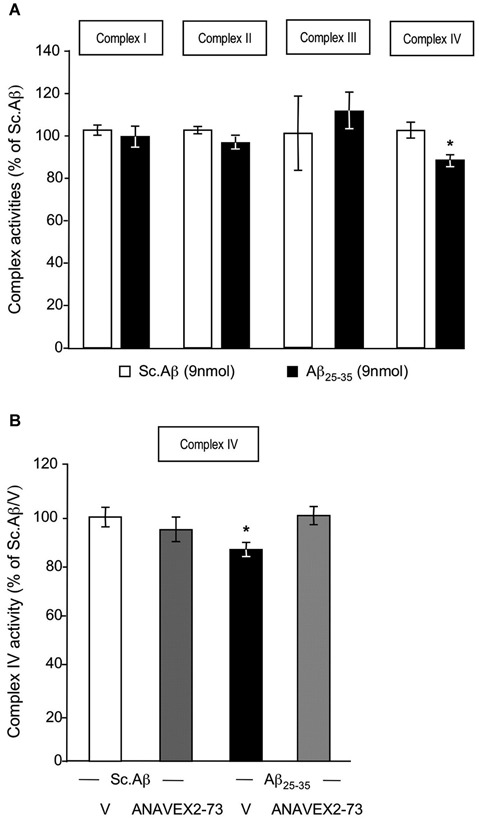
**ANAVEX2-73 protected against Aβ_25–35_-induced alteration of complex IV activity**. Mitochondria were loaded in the spectrophotometer cuvette with appropriate buffer at 30°C and variations of absorption were recorded after addition of specific substrates. **(A)** Effect of Aβ_25–35_ ICV injection on complex activities (% of Sc. Aβ) *n* = 8–9 per group, * *p* < 0.05 vs. Sc. Aβ Student’s *t*-test. **(B)** Mice were treated with ANAVEX2-73 (0.3 mg/kg IP) or vehicle, before injection of Sc. Aβ or Aβ_25–35_ (9 nmol ICV). Complex IV activity was measured after 7 days (% of Sc. Aβ). *n* = 5–9 per group, *F*_(3,26)_ = 3.00, *p* < 0.05. * *p* < 0.05 vs. Sc. Aβ; Dunnett’s test.

We analyzed the nature of the Aβ_25–35_-induced alteration of respiration (Figure 4). The measure of each complex activity showed that only the complex IV activity was significantly decreased by −14% (Figure 4A). The ANAVEX2-73 treatment prevented the Aβ_25–35_-induced decrease in complex IV activity (Figure 4B).

**Figure 5:**
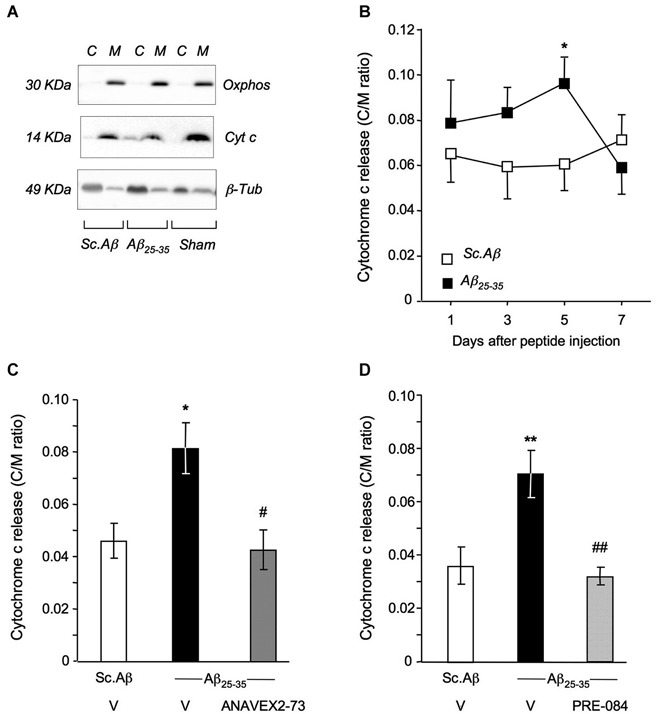
**ANAVEX2-73 and PRE-084 prevented Aβ_25–35_-induced cytochrome c release. (A)** Typical blots of mitochondrial (M) and cytosolic (C) fractions with cytochrome *c*, oxphos and β-tubulin antibodies. **(B)** Cytochrome c content (cytosol to mitochondrial content ratios). Mice were injected with Sc. Aβ or Aβ_25–35_ (9 nmol ICV) and sacrificed at indicated days. *n* = 9–12 per group. * *p* < 0.05 vs. Sc. Aβ. Student’s *t*-test. **(C, D)** Mice were treated with ANAVEX2-73 (0.3 mg/kg IP) or PRE-084 (0.5 mg/kg IP) before Aβ_25–35_ and sacrificed after 5 days. *n* = 8–17, *F*_(2,37)_ = 5.96, *p* < 0.01 in **(C)**; *n* = 8–14, *F*_(2,33)_ = 8.19, *p* < 0.01 in **(D)**. ^*^p < 0.05, ^**^p < 0.01 vs. Sc. Aβ, ^#^*p* < 0.05, ^##^*p* < 0.01 vs. Aβ_25–35_; Dunnett’s test.

Among the different biochemical markers that can be examined to evidence the damage resulting from impaired mitochondrial functionality, we first addressed cytochrome *c* release from the mitochondrial membrane into the cytosol (Figure 5A). The time-course analysis showed that significant cytochrome *c* release can be evidenced at day 5 after the peptide injection (Figure 5B). At this timepoint, both the ANAVEX2-73 treatment (Figure 5C) and PRE-084 treatment (Figure 5D) completely prevented the Aβ_25–35_-induced cytochrome *c* release in the mouse hippocampus.

**Figure 6:**
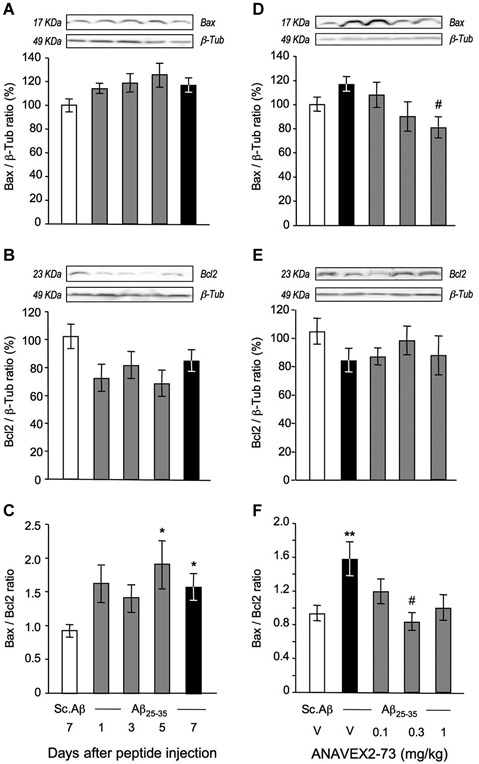
**ANAVEX2-73 protected against Aβ_25–35_-induced increase in Bax/Bcl-2 ratio in the mouse hippocampus. (A–C)** Mice were sacrificed at 1, 3, 5 and 7 days after Aβ_25–35_ injection and Bax and Bcl-2 contents determined by western blots. **(D–F)** Effect of ANAVEX2-73 (0.1–1 mg/kg IP) on Bax and Bcl-2 levels, 7 days after Aβ_25–35_ injection. Typical blots are represented above the bar graphs. Mice were injected with ANAVEX2-73 or vehicle before Aβ_25–35_ peptide (9 nmol). *n* = 5–15 per groups. *F*_(4,44)_ = 1.98, *p* > 0.05 in **(A)** ; *F*_(4,44)_ = 1.95, *p* > 0.05 in **(B)** ; *F*_(4,44)_ = 2.66, *p* < 0.05 in **(C)**;* F*_(4,49)_= 3.01, *p* < 0.05 in **(D)**, *F*_(4,49)_ = 0.89, *p* > 0.05 in **(E)**, *F*_(4,49)_ = 3.09, *p* < 0.05 in **(F)**. ^*^p < 0.05, ^**^p < 0.01 vs. Sc. Aβ; ^#^*p* < 0.05 vs. Aβ_25–35_; Dunnett’s test.

We then measured Bax and Bcl-2 protein expression, by a western blot approach, in order to confirm that the impact of the treatment on mitochondrial damage readily transfer into anti-apoptosis protection (Figure 6). The Aβ_25–35_ treatment resulted, between 1 to 7 days after injection, in a progressive but non-significant increase in Bax level in the hippocampus (Figure 6A), accompanied by a trend to diminution of Bcl-2 level (Figure 6B). As a consequence, the Bax/Bcl-2 ratio increased, and significantly at days 5 and 7 after the peptide injection (Figure 5C). ANAVEX2-73 was tested at 0.1–1 mg/kg. A dose-dependent decrease in Bax level was measured at the highest dose tested (Figure 6D). The drug impacted marginally Bcl-2 levels (Figure 6E), but the data examined as Bax/Bcl-2 ratio clearly showed a beneficial effect of the compound, particularly significant at 0.3 mg/kg (Figure 6F).

## Discussion

In this study, we showed that mitochondrial functionality was altered several days after an ICV injection of the Aβ_25–35_ peptide in mouse. It was shown by reduced respiration rates, decreased complex IV activity and increased oxidative stress and proapoptotic markers. Five days after a single injection of oligomeric Aβ_25–35_ peptide, respiration was reduced in isolated mitochondria from mouse hippocampus, as compared with control Sc. Aβ-treated mice. Basal respiration (State 2 and State 4) was unaffected by Aβ_25–35_, but ADP-stimulated respiration (State 3) was significantly decreased. Reduced State 3 respiration resulted in a decreased control ratio in Aβ_25–35_-injected mice. Uncoupled state was also altered, suggesting a defect in respiratory chain complex I-IV rather than a defect in ATP synthase complex activity. The reduced respiratory rates and control ratio observed in this *in vivo* model are coherent with previous studies exploring the impact of Aβ peptides, and particularly Aβ_25–35_, on mitochondrial respiration *in vitro*. Application of Aβ_25–35_ on isolated mitochondria decreased respiration rates (Casley et al., 2002a; Aleardi et al., 2005; Clementi et al., 2005). Interestingly, in these studies, basal respiration (State 4) was also altered after Aβ_25–35_ treatment. In the same way, mitochondria isolated from cultured neurons and exposed to Aβ_25–35_ showed decreased ATP production, suggesting a reduced mitochondrial respiration (Casley et al., 2002b; Dong et al., 2010). In search for an explanation to this decreased mitochondrial respiration after Aβ_25–35_, we measured the respiratory chain complex I-IV activities. We found that complex IV activity was significantly reduced 5 days after Aβ_25–35_. In most of the *in vitro* studies, application of Aβ_25–35_ on cultured neurons or directly on isolated mitochondria resulted in reduced activities of complexes I and IV (Casley et al., 2002a,b; Aleardi et al., 2005; Clementi et al., 2005; Dong et al., 2010). This appears to be a difference between *in vitro* and *in vivo* studies with the Aβ_25–35_ peptide. It could explain the decreased state 4 respiration observed *in vitro*. But, consistent with our results, are data in transgenic mice expressing human mutant amyloid precursor protein (hAPPm). Aβ peptide has been found to accumulate in mitochondria and respiratory rates and complex IV activity were also decreased in these animals (Caspersen et al., 2005; Rhein et al., 2009; Du et al., 2010). Moreover, Canevari et al. (1999) described a specific and concentration-dependent decrease in complex IV activity after Aβ_25–35_ application in isolated mitochondria. Disruption of complex IV activity induced by Aβ peptides could be due to a direct interaction between Aβ and the enzymatic subunits (Crouch et al., 2006; Hernandez-Zimbron et al., 2012) but also to the direct induction of oxidative stress by Aβ peptides, and particularly Aβ_25–35_. Indeed, membrane lipid peroxidation induced by ROS overproduction might alter complex IV subunits and disrupt complex IV activity (Paradies et al., 1998; Bobba et al., 2013), as we confirmed the marked increase in oxidative stress in mouse hippocampus, already described in this model (Meunier et al., 2006). The release of cytochrome *c* from mitochondria into the cytosol that was increased 5 days after Aβ_25–35_ injection can also contribute to the dysfunction of the mitochondrial respiratory chain and thus to the oxidative stress. Concomitantly to the cytochrome *c* release, the ratio Bax/Bcl-2 was significantly increased in mouse hippocampus, 5 and 7 days after Aβ_25–35_ injection and this is likely related to the cellular response to the high level of oxidative stress and decreased mitochondrial respiration (Mattson et al., 2008).

The aim of the study was to establish whether the localization of the σ_1_R on mitochondria (Klouz et al., 2002) and more precisely at MAM (Hayashi and Su, 2007) is related to its particular neuroprotective activity. Indeed, both ANAVEX2-73 and PRE-084, prevented Aβ_25–35_-induced oxidative stress and increase in pro-apototic markers. The results are coherent with previously reported observations, since the σ_1_R has been involved in regulation of oxidative stress (Pal et al., 2012; Tsai et al., 2012). Moreover, σ_1_R agonists prevented oxidative stress-induced apoptosis *in vitro* through upregulation of the anti-apoptotic Bcl-2 protein expression (Meunier and Hayashi, 2010). The σ_1_R activation also prevented Bax increase and caspase-3 activation, linked to cytochrome *c* release, after glutamate insult in retinal ganglional cells (Tchedre and Yorio, 2008). Another study also showed a decreased caspase-3 activation after PRE-084 treatment in a Huntington’s disease model (Hyrskyluoto et al., 2013). ANAVEX2-73 and PRE-084 both fully reversed state 3 and uncoupled state respiration deficits. Surprisingly both drugs also increased basal respiration (state 2 and state 4) and PRE-084, but not ANAVEX2-73 increased state 3 and uncoupled state respiration, compared to control mice. These differences in ANAVEX2-73 and PRE-084 effects could be explained by the higher affinity of the later for the σ_1_ receptor. Although state 3 respiration raised to the control level after ANAVEX2-73 treatment, CR was not different from the Aβ_25–35_ group because state 4 respiration also increased. However, the high increased of both state 3 and state 4 respiration after PRE-084 treatment increased the control ratio to the control level. Such increased respiration after stimulation of σ_1_R has never been described and is not observed in Sc. Aβ-treated control mice. An attempt to explain this phenomenon is challenging. The increased state 3 and uncoupled state respiration rates observed after PRE-084 treatment were clearly not an artifact or due to experimental conditions as all the samples were prepared in strictly the same way and measures realized randomly among treatment groups and in several experimental sessions. Few studies have examined the involvement of σ_1_R in mitochondrial respiration. BHDP is a selective ligand with nanomolar affinity for the σ_1_ receptor (Klouz et al., 2003). A three days pretreatment with BHDP in rats prevented the decrease in CR in mitochondria isolated from rat liver after ischemia-reperfusion (Klouz et al., 2008). We previously suggested that protective effect on mitochondrial function of σ_1_ agonist could be attributable to the presence of σ_1_R to the mitochondrial membrane (Klouz et al., 2002). Indeed, σ_1_R are present in lipid rafts and modify their composition and particularly the ganglioside content (Hayashi and Su, 2005). In mitochondrial membrane, lipid rafts microdomains, and particularly the glycosphingolipid GD3, are involved in alteration of mitochondrial membrane potential and apoptosis (Malorni et al., 2007). Therefore, an explanation of increased mitochondrial respiration by σ_1_ ligand could be through the capacity of the protein to modulate lipid rafts gangliosids at mitochondrial membrane. Another explanation could be the chaperone activity of the σ_1_ protein on InsP_3_ receptor at the ER membrane (Hayashi and Su, 2007). The InsP_3_ receptor stabilization allows proper Ca^2+^ exchange between ER and mitochondria, which are critical for mitochondrial bioenergetics (Cárdenas et al., 2010). Cutamesine (SA4503), another selective σ_1_ agonist, increased ATP production in cardiomyocytes, even without pathological conditions, suggesting an increase in mitochondrial respiration after σ_1_R stimulation (Tagashira et al., 2013). The increased mitochondrial bioenergetics after σ_1_ agonist treatment is largely attribuable to ER Ca^2+^ mobilization into mitochondria, because treatment with xestospongin C, an antagonist of the InsP_3_ receptor abolished the effects of the σ_1_ agonist on ATP production. However, in our model, this mechanism did not seem to be involved in the σ_1_ protein-induced increased mitochondrial respiration because the agonist was injected only once, five days before the mitochondrial isolation. Moreover, Tsai et al. (2009) described Ca^2+^-independent perturbations of mitochondrial functions in hippocampal neurons after σ_1_ receptor knockdown with siRNA. To note, another interesting consequence of σ_1_R activity is the modulation of different genes expression. Some of them are involved in oxidative stress regulation, *e.g*., NAD(P)H quinone oxidoreductase (Nqo1), Superoxide dismutase (SOD), Atf-4 and Prdx6 (Pal et al., 2012; Tsai et al., 2012). It could be worth investigating whether the PRE-084-induced increased respiratory efficacy could be due to the modification of expression of specific genes.

In conclusion, we provided here the clear evidence that the neuroprotective activity of σ_1_ receptor agonists, including the mixed muscarinic ligand/σ_1_ agonist ANAVEX2-73, involves mitochondrial protection in AD models. Indeed, the drug protected mitochondria against Aβ_25–35_ insult. Mitochondrial dysfunction in AD is a critical hallmark of the pathology, as mitochondria are, at the same time, a target and promoter of the physiopathological processus (for review, see Eckert et al., 2010; Selfridge et al., 2013). Mitochondrial dysfunction and, notably, resulting oxidative stress are responsible for the increased Aβ production (Dyrks et al., 1992; Leuner et al., 2007) and Tau hyperphosphorylation (Melov et al., 2007), the two biochemical signatures of AD. Thus, by preserving mitochondrial function ANAVEX2-73 could expectedly be more than a symptomatic drug and block the physiopathological amplification of the pathology.

## Conflict of interest statement

Valentine Lahmy and Vanessa Villard are employees of Amylgen. Tangui Maurice is a member of the scientific advisory board of Anavex Life Sciences and scientific director of Amylgen. The companies had no role in the present research, except providing drug and funding. Other authors declare no conflict of interest.
